# Evaluation of the effectiveness of using capecitabine versus capecitabine combined with oxaliplatin during preoperative radiotherapy for patients with rectal cancer: A retrospective cohort study

**DOI:** 10.1097/MD.0000000000041580

**Published:** 2025-02-21

**Authors:** Qiang Zuo, Wen Wang, Qiang Chen, Miao Wu

**Affiliations:** a Department of Gastrointestinal Hernia, The Second People’s Hospital of Yibin City, Yibin, China; b Department of Prevention and Control of Chronic Noncommunicable Diseases, Yibin Center for Disease Control and Prevention, Yibin, China.

**Keywords:** capecitabine, neoadjuvant chemoradiotherapy, oxaliplatin, rectal cancer

## Abstract

The purpose of this study was to assess and compare the clinical effectiveness of capecitabine monotherapy and that of capecitabine combined with oxaliplatin as neoadjuvant chemoradiotherapy during preoperative radiotherapy in the management of low and middle rectal cancer. A retrospective cohort study was performed. Medical data were collected from individuals with locally progressing low and middle rectal cancer admitted to a regional hospital in China. Two groups of patients were formed for different chemoradiotherapy regimens: the oxaliplatin group and the capecitabine monotherapy group. Within the oxaliplatin group, the CAPEOX regimen was applied for 2 rounds during radiotherapy, intravenous infusion of oxaliplatin was administered 1 day prior to radiotherapy. In the capecitabine monotherapy group, capecitabine was implemented once daily during radiotherapy, and no medication was taken without radiotherapy. A total of 260 patients were included in the study. When oxaliplatin is administered concurrently with preoperative radiation therapy for patients with locally progressing low and middle rectal cancer, the pathologic complete remission rate can be considerably increased without appreciably increasing adverse effects or impairing postoperative recovery. On the other hand, the long-term effectiveness against metastasis and/or recurrence showed no discernible benefit.

## 1. Introduction

Globally, colorectal cancer ranks second in mortality and third in morbidity, and it primarily affects younger individuals, who are often already in the middle or late stage at diagnosis.^[1]^ Treatment options may differ depending on the anatomical location of the tumor; however, the treatment approaches for colon cancer and upper rectal carcinoma are similar. Patients with locally progressing (cT3/cT4 or N+) low and middle rectal carcinoma are treated with radical surgery and postoperative adjuvant chemotherapy; preoperative neoadjuvant chemoradiotherapy and postoperative adjuvant chemotherapy; or total neoadjuvant therapy (TNT) followed by radical surgery.^[2–4]^ Neoadjuvant chemoradiotherapy can successfully postpone local recurrence and distant metastasis, decrease local recurrence, increase the chance of surgical anal preservation, and even lead to the selection of nonsurgical observation strategies after clinical complete remission (cCR).^[5–7]^ For individuals with low and intermediate levels of rectal cancer, radical surgery inevitably poses the risk of surgery-related complications, including low anterior resection syndrome, anastomotic leakage, and genitourinary system dysfunction, and may even lead to permanent stoma, which significantly impairs patients’ quality of life.^[8]^ The safety and efficacy of nonsurgical treatment after achieving cCR through neoadjuvant chemoradiotherapy have been confirmed,^[9]^ and this approach enables some patients to avoid surgical trauma and related complications while preserving organs and improving quality of life are possible. Intensified systemic chemotherapy before surgery could improve the pathologic complete remission (pCR) rate and provide survival benefits. However, the best mode of neoadjuvant therapy is unclear.^[10,11]^ It has been debated whether adding oxaliplatin with 5-fluorouracil or capecitabine concurrently with chemoradiotherapy increases pCR rates and extends survival. Rödel et al^[12]^ and Deng et al^[13]^ reported that the pCR rate was considerably increased by the addition of oxaliplatin. However, according to other studies, adding oxaliplatin did not increase either survival or the pCR rate.^[14–18]^

In order for more patients to avoid surgical trauma and related complications, it is necessary to continue to explore new adjuvant therapy schemes to improve the pCR rate. In this retrospective analysis, we examined the clinicopathologic information of 260 individuals with locally progressing intermediate and low rectal cancer (within 10 cm of the anal border) admitted to our hospital in Yibin city from February 2019 to March 2023, with the aim of comparing the clinical efficacy of 2 different neoadjuvant chemoradiotherapy protocols.

## 2. Materials and methods

### 2.1. Study design and setting

**Figure 1.**
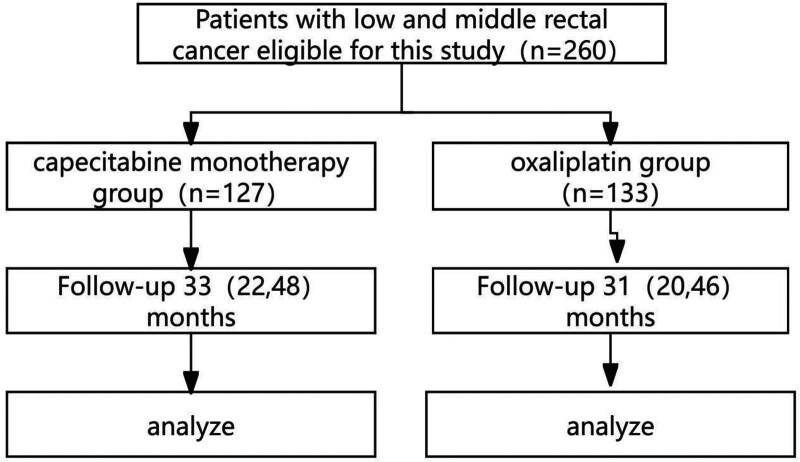
Study flow diagram.

We performed a retrospective cohort study. Medical data were collected from individuals with locally progressing low and middle rectal cancer admitted to our hospital from February 2019 to March 2023. Two groups of patients were formed for different chemoradiotherapy regimens: the oxaliplatin group and the capecitabine monotherapy group. A total of 260 patients, 164 men and 96 women aged 26 to 75 years, with an average age of 59 years, were included. Preoperative neoadjuvant therapy was completed in both groups. Adverse reactions during neoadjuvant chemoradiotherapy were classified with reference to the Classification Standard of Common Toxic and Side Effects of Anticancer Drugs. The tumors were staged via the American Cancer Society 7th Edition TNM staging system. The Ethics Committee approved this project (Fig. 1).

### 2.2. Inclusion and exclusion criteria

#### 2.2.1. Inclusion criteria

Pathology biopsy confirming rectal adenocarcinoma prior to treatment; diagnosis with stage II to III disease (T3/T4 or N+) based on the 7th edition of the American Joint Committee on Cancer criteria as assessed by pelvic magnetic resonance examination; distance of <10 cm between the rectal tumor and the anal boundary; and completed neoadjuvant therapy with complete clinicopathological data.

#### 2.2.2. Exclusion criteria

Stage I or IV clinical rectal cancer prior to therapy; anal margin and lower margin of the rectal tumor broken by more than 10 cm; failure to complete neoadjuvant therapy; age > 75 years or serious heart or lung disease before treatment; absence of clinicopathological data; and patients who could not be contacted at follow-up and whose prognosis was unknown.

### 2.3. Neoadjuvant chemoradiotherapy regimen

Within the oxaliplatin group, intravenous infusion of oxaliplatin (130 mg/m^2^) was administered 1 day prior to radiotherapy. Thereafter, capecitabine (1000 mg/m^2^) was ingested twice daily for 2 weeks following treatment, the medication was discontinued for 1 week, and 2 cycles were performed during radiotherapy. For a total of 25 cycles, a single daily dose of radiation therapy was given 5 times a week with a 45 Gy clinical target area. The medication was discontinued for 2 weeks after radiotherapy; then, the CAPEOX regimen was applied for 3 rounds of chemotherapy. Surgery was performed approximately 12 weeks after radiotherapy ended. In the single-drug capecitabine group, capecitabine + CRT and radiotherapy were also implemented once daily, 5 times weekly, a total of 25 times, with a clinical target area of 45 Gy. On the day of radiotherapy, 825 mg/m^2^ capecitabine was taken orally. No medication was taken without radiotherapy. The medication was discontinued for 2 weeks after radiotherapy, and the CAPEOX regimen was applied for 3 rounds of chemotherapy. Surgery was performed approximately 12 weeks after radiotherapy ended.

### 2.4. Observed indicators

The 2 groups’ overall general parameters, including sex, age, body mass index (BMI), tumor location, clinical stage before and after neoadjuvant therapy, pathological stage, adverse reaction grade during chemoradiotherapy, mrTRG and follow-up status, were recorded. The values for mrTRG4 and mrTRG5 were very close, so they were combined. The main outcome measures of the 2 groups included the pCR, local recurrence rate and/or distant metastasis rate.

### 2.5. Follow-up

Records of any distant metastases or local recurrences in the capecitabine monotherapy group at 33 (22, 48) months and in the oxaliplatin group at 31 (20, 46) months were obtained through telephone calls and outpatient follow-up until May 2024.

### 2.6. Analytical statistics

SPSS 22.0 was used for statistical analysis. The measurement data were normally distributed and are shown as (*x* ± *s*). The Mann–Whitney *U* test was employed to contrast the 2 sets. Independent sample t tests were employed to represent comparisons between the 2 groups. *M* (IQR) was employed to represent data that were not normally distributed. For intragroup comparisons, the Wilcoxon signed rank test was employed. The chi-square test was used to compare data between groups, and the Mann–Whitney *U* test was used to compare data between grades. Counting data are shown as n (%). *P* < .05 was the threshold for statistical significance at the test level.

## 3. Results

### 3.1. Particulars and broad details

In total, the 2 groups included 260 patients who were 58.65 ± 10.27 years old, among which 127 patients in the capecitabine monotherapy group were 58.98 ± 9.32 years old, and 79 (62.2%) were males. Among the patients in the oxaliplatin group, 133 patients were 58.34 ± 11.12 years old, and 85 (63.9%) were males. No statistically significant differences in BMI, height, weight, age, sex, or tumor distance from the anal margin were detected between the 2 sets (*P* > .05), indicating comparability (Table 1).

**Table 1:** Comparison of the 2 groups’ general data.

	Capecitabine monotherapy (n = 127)	Oxaliplatin (n = 133)	*X*^2^/*t*/*z*	*P*
Age (yr)	58.98 ± 9.32	58.34 ± 11.12	0.506	.613*
Sex (%)			0.081	.776***
Male	79 (62.20)	85 (63.91)		
Female	48 (37.80)	48 (36.09)		
Height (m)	1.61 ± 0.07	1.59 ± 0.08	1.249	.213*
Weight (kg)	59.92 ± 9.26	58.5 ± 10.43	1.158	.248*
BMI (kg/m^2^)	23.05 (20.61, 25.46)	23.03 (20.2, 25.47)	−0.624	.532**
BSA	1.59 ± 0.14	1.57 ± 0.15	1.398	.163*
Hypertension (%)			0.022	.881***
Yes	18 (14.17)	18 (13.53)		
No	109 (85.83)	115 (86.47)		
T2DM (%)			0.086	.770***
Yes	3 (2.36)	5 (3.76)		
No	124 (97.64)	128 (96.24)		
T stage (%)			−0.839	.401**
T1	0 (0)	0 (0)		
T2	4 (3.15)	0 (0)		
T3	89 (70.08)	94 (70.68)		
T4	34 (26.77)	39 (29.32)		
N stage (%)			−1.591	.112**
N0	34 (26.77)	44 (33.08)		
N1	61 (48.03)	66 (49.62)		
N2	32 (25.2)	23 (17.29)		
N3	0 (0)	0 (0)		
M stage (%)			–	–
M0	127 (100)	133 (100.0)		
M1	0 (0)	0 (0)		
CRM (%)			2.567	.109***
Positive	68 (53.54)	58 (43.61)		
Negative	59 (46.46)	75 (56.39)		
Vascular nerves (%)			1.973	.160***
Yes	15 (11.81)	9 (6.77)		
No	112 (88.19)	124 (93.23)		
Distance from anal margin	5 (3.5, 6)	5 (3, 7)	−0.944	.345**
Specimen lymph node	7.30 ± 3.70	7.40 ± 4.00	−0.219	.827*

BSA = body surface area, T2DM = type 2 diabetes.

* Represents the *t* test.

** Represents the Mann–Whitney *U* test.

*** Represents the chi-square test.

### 3.2. Preoperative chemoradiotherapy

Patients in both cohorts finished all preoperative chemotherapy and radiation treatments. Patients in both groups reported different degrees of discomfort during treatment, and most patients had grade I to II adverse reactions. There was no significant difference in side effects between the 2 groups during therapy (*P* = .61; Table 2).

**Table 2:** Adverse reactions during neoadjuvant therapy.

Group	Cases	Classification of adverse reactions					*z*	*P*
		0	I	II	III	IV		
Capecitabine monotherapy	127	28 (22.05)	49 (38.58)	38 (29.92)	10 (7.87)	2 (1.57)	−0.516	.606
Oxaliplatin	133	43 (32.33)	35 (26.32)	38 (28.57)	14 (10.53)	3 (2.26)		

### 3.3. Tumor regression after neoadjuvant therapy

After treatment, the tumor regression grades were evaluated by magnetic resonance imaging combined with a digital rectal examination and colonoscopy. The condition of most patients improved, and the tumors obviously regressed. The magnetic resonance imaging tumor regression grade (mrTRG) did not significantly differ between the 2 groups (*P* = .18; Table 3).

**Table 3:** Comparison of tumor regression after neoadjuvant therapy between the 2 groups.

	Capecitabine monotherapy (n = 127)	Oxaliplatin (n = 133)	*X* ^2^	*P*
mrTRG classification (%)			−1.332	.183
mrTRG1	23 (18.11)	34 (25.56)		
mrTRG2	61 (48.03)	36 (27.07)		
mrTRG3	34 (26.77)	38 (28.57)		
mrTRG4,5	9 (7.09)	25 (18.8)		

mrTRG = magnetic resonance imaging tumour regression grade.

### 3.4. Postoperative recovery

In both groups, the time until leaving bed, duration of intestinal function recovery, and duration of liquid diet were the second postoperative days for most patients, and the duration of hospitalization for most patients was 6 days. The 2 groups did not differ significantly in terms of postoperative recovery (*P* = .31; *P* = .95; *P* = .06; *P* = .10; Table 4).

**Table 4:** Postoperative recovery.

	Capecitabine monotherapy	Oxaliplatin	*z*	*P*
Time of first postoperative exhaust defecation (d)	2 (1, 2)	2 (1, 2)	−0.066	.947
Time of first liquid diet after surgery (d)	2 (1, 2)	2 (1, 3)	−1.858	.063
Time of getting out of bed for the first time following surgery (d)	2 (2, 3)	2 (2, 3)	−1.007	.314
Postoperative hospital stay (d)	6 (5, 7)	6 (5, 7)	−0.006	.995

### 3.5. Postoperative pathological complete remission

pCR was interpreted as the absence of residual tumor cells in the radical excision specimen. Postoperative pathological results revealed that 23 patients in the capecitabine group had no residual tumor cells, which made the pCR rate 18.1%. In contrast, 39 patients in the oxaliplatin group achieved pCR, which made the pCR rate 29.3%. The pCR rate in the oxaliplatin group was greater than that in the capecitabine monotherapy group, and the difference was significant (*P* = .03; Table 5).

**Table 5:** Comparison of pCR rates between the 2 groups.

Group	Cases	pCR rates (%)		*X* ^2^	*P*
		No	Yes		
Capecitabine monotherapy	127	104 (81.89)	23 (18.11)	4.498	.034
Oxaliplatin	133	94 (70.68)	39 (29.32)		

pCR = pathologic complete remission.

### 3.6. Recurrence and metastasis

Recurrence or metastasis was defined as the cancer having been confirmed by imaging, endoscopic biopsy, needle biopsy, and immunohistochemistry at follow-up. 32 patients (24.4%) in the capecitabine group and 33 patients (24.8%) in the oxaliplatin group developed recurrence and/or metastasis during the follow-up period; however, there was no significant difference in these outcomes between the 2 groups (*P* = .94; Table 6).

**Table 6:** Comparison of recurrence and/or metastasis rates during follow-up between the 2 groups.

Group	Cases	Recurrence or metastasis (%)		*X* ^2^	*P*
		No	Yes		
Capecitabine monotherapy	127	96 (75.6)	31 (24.4)	0.006	.940
Oxaliplatin	133	100 (75.2)	33 (24.8)		

## 4. Discussion

In this study, we found that increasing the intensity of systemic chemotherapy during neoadjuvant therapy did not increase the degree of adverse reactions but did increase the pCR rate. However, increasing the intensity of preoperative systemic chemotherapy did not improve patients’ long-term prognosis.

At present, for T3-4/N+ low and middle rectal cancer patients, the standard mode of preoperative chemoradiotherapy in China is preoperative long-term chemoradiotherapy (oral capecitabine monotherapy at the same time as radiotherapy, 6–8 weeks after radiotherapy) or preoperative short-term radiotherapy (operation within 1 week after radiotherapy). However, the complete remission rate of this treatment mode is not satisfactory, and this standard of care needs to be optimized. Possible optimization schemes include increasing the radiotherapy dose;^[19]^ increasing the intensity of systemic chemotherapy;^[11,20]^ lengthening the time interval from radiotherapy to surgery;^[21]^ incorporating combination chemotherapy;^[22]^ and performing full neoadjuvant chemoradiotherapy (TNT).^[23]^ Usually, the intensity of systemic chemotherapy is increased, most commonly by administering 2-drug combination chemotherapy with fluorouracil or capecitabine combined mainly with oxaliplatin and irinotecan.^[20,24]^ Currently, the use of targeted drugs in combination with radiotherapy is not recommended. Many clinical studies on oxaliplatin have been conducted, but the results are inconsistent. According to certain studies, the addition of oxaliplatin to preoperative radiation therapy can greatly increase the pCR rate.^[11,25]^ However, other studies have refuted this finding, demonstrating that the addition of oxaliplatin to preoperative radiation increases toxicity and adverse effects without increasing the pCR rate or significantly improving the long-term prognosis.^[26,27]^ There are numerous chemotherapy options during radiotherapy, such as oral capecitabine alone and oral capecitabine combined with 2 drugs for 1 cycle or 2 cycles. More studies are needed to determine which method is best.

Through retrospective analysis of the effectiveness of various neoadjuvant therapy regimens in 2 medical groups, this study confirmed that administering 2 cycles of oxaliplatin concurrently with preoperative radiotherapy increased the pCR rate from 18.1% to 29.3% but did not significantly increase adverse reactions or affect postoperative recovery. However, there was no appreciable benefit in terms of long-term effectiveness against metastasis and recurrence. Our findings are consistent with the method of increasing pCR rate, increasing the intensity of systemic chemotherapy. Individuals with low and middle rectal cancer who undergo radical surgery are unavoidably exposed to hazards such as anastomotic leakage, syndrome of low anterior resection, dysfunction of the genitourinary system, and even stomostomy. These consequences can have a negative impact on patients’ quality of life. The “watching and waiting” strategy for patients who achieve cCR through nonsurgical treatment has been shown to be safe and reliable.^[28–30]^ A follow-up study revealed that 25.2% of patients with nonoperative cCR had local recurrence, 8.0% had distant metastasis, and most of the patients experienced recurrence and metastasis within 2 years.^[2]^ For patients with local recurrence, timely remedial radical surgery does not affect the oncological prognosis. Therefore, maximizing cCR through preoperative treatment is a key goal of the current research.

Many people are focused on improving the pCR rate, and some people have even tried using multi-drug combinations (oxaliplatin + irinotecan + fluorouracil) with concurrent chemoradiotherapy; however, these regimens seem to have had no significant impact on the pCR rate, and they may even increase the occurrence of toxic side effects, making patients unable to tolerate them. Currently, the TNT regimen is favored by clinicians, including the induction TNT and consolidation TNT, but which TNT regimen is best remains unclear.^[31,32]^ The neoadjuvant radiotherapy and chemotherapy regimen in this study yielded a pCR rate very similar to that of the current TNT regimen, and as individualized treatment becomes more common, it is unlikely that only 1 neoadjuvant treatment regimen is considered the best. The authors believe that the new adjuvant therapy regimen used in this study can achieve pCR rates similar to those of TNT, so it is feasible for most patients to abandon TNT, as TNT may lead to a decrease in tolerance for surgery in some patients. Alternatively, introducing 2 cycles of CAPEOX as a consolidation TNT regimen during radiotherapy alongside TNT may yield better outcomes, and this regiment is worth investigating further. Currently, the combination of immunotherapy with neoadjuvant chemoradiotherapy is being actively researched, and it is believed that the neoadjuvant treatment regimen for lower and middle rectal cancer will become increasingly precise, safe, and effective in the future.

### 4.1. Limitations

Our study is a retrospective analysis, so there is some selection bias. Some of the data were obtained from electronic medical records, which may cause information bias. Owing to the single-center nature of the study, an insufficient sample size was used. On the other hand, in the follow-up process, some patients lost to follow up, resulting in the loss of access bias; we reduced the loss of follow-up bias by removing patients with similar outcomes. Large-scale trials are still needed before definitive conclusions can be drawn.

## 5. Conclusion

For patients with locally progressed low and middle rectal cancer, the administration of oxaliplatin at the same time as preoperative radiotherapy can significantly improve the pCR rate without significantly increasing adverse reactions or significantly affecting postoperative recovery. However, there was no obvious advantage in long-term efficacy against recurrence and metastasis. Further research is needed to determine which preoperative treatment is optimal. Additionally, with today’s emphasis on individualized treatment, more research is needed to determine how to select the most appropriate neoadjuvant treatment for each patient.

## Acknowledgments

The authors thank all the patients at Department of Gastrointestinal Hernia, the Second People’s Hospital of Yibin City. We thank Andy Brandt, PhD for editing drafts of this manuscript (https://china.aje.com/api/certificate/1E87-7072-359B-BA9B-589A/pdf).

## Author contributions

**Conceptualization:** Qiang Zuo, Miao Wu.

**Data curation:** Qiang Zuo, Qiang Chen.

**Formal analysis:** Qiang Zuo, Wen Wang.

**Investigation:** Miao Wu.

**Writing – original draft:** Qiang Zuo.
